# Inguinoscrotal Hernia of the Urinary Bladder

**DOI:** 10.7759/cureus.56636

**Published:** 2024-03-21

**Authors:** Shravani Sripathi, Najiha Farooqi, Mohamed K Kamel, Rikat Baroody, Akram Alashari

**Affiliations:** 1 Department of General Surgery, Central Michigan University (CMU) Medical Education Partners, Saginaw, USA

**Keywords:** scrotal hernia, urinary bladder hernia, inguinal hernia, cystocele, hernia

## Abstract

Inguinal bladder hernia (IBH) is a rare clinical condition that may present as scrotal swelling. Most patients are asymptomatic and found incidentally at the time of herniorrhaphy. IBH continues to pose a challenge to surgeons before, during, and even after herniorrhaphy. This case report aims to describe the case of the incarcerated right inguinal hernia containing the small bowel and the urinary bladder herniation. An 81-year-old male presented to the emergency department with complaints of abdominal pain, distension, and swelling in the right groin. Physical examination was remarkable for incarcerated right inguinal hernia with tenderness to palpation. A CT scan demonstrated a right inguinal hernia containing a small bowel. The urinary bladder was noted to be adherent to the hernia sac. The hernia sac and urinary bladder were reduced, and Lichtenstein tension-free hernia repair was performed. The postoperative course was uneventful without any complications. IBHs are uncommon. Unrecognized bladder hernias can cause bladder injury during surgery. It is particularly common in individuals with long-standing hernias and should be anticipated during surgery. High-risk patients including obese, older men, who have urinary symptoms that need further evaluation with a CT scan, ultrasound, or cystography to prevent iatrogenic injury and complications. Management consists of reduction or resection of the herniated bladder followed by hernia repair.

## Introduction

Inguinal bladder hernia (IBH), first described by Levine in 1951 as a “scrotal cystocele,” is a rare clinical condition [1]. The incidence of bladder involvement in inguinal hernias is 1%-4% [2]. Bladder hernias most commonly present as direct inguinal hernias, frequently occurring on the right side with a 70% male predominance [3]. The incidence of IBHs in men over age 50 years may be as high as 10% [2]. Large bladder hernias are almost always associated with voiding symptoms. However, most cases are asymptomatic. Preoperatively, less than 7% of bladder hernias are found, 16% are discovered after surgery because of complications, and the rest, perioperatively [4]. IBH continues to pose a challenge to surgeons before, during, and even after hernia repair. We present a case of an incarcerated right inguinal hernia containing a small bowel with urinary bladder herniation.

## Case presentation

**Figure 1:**
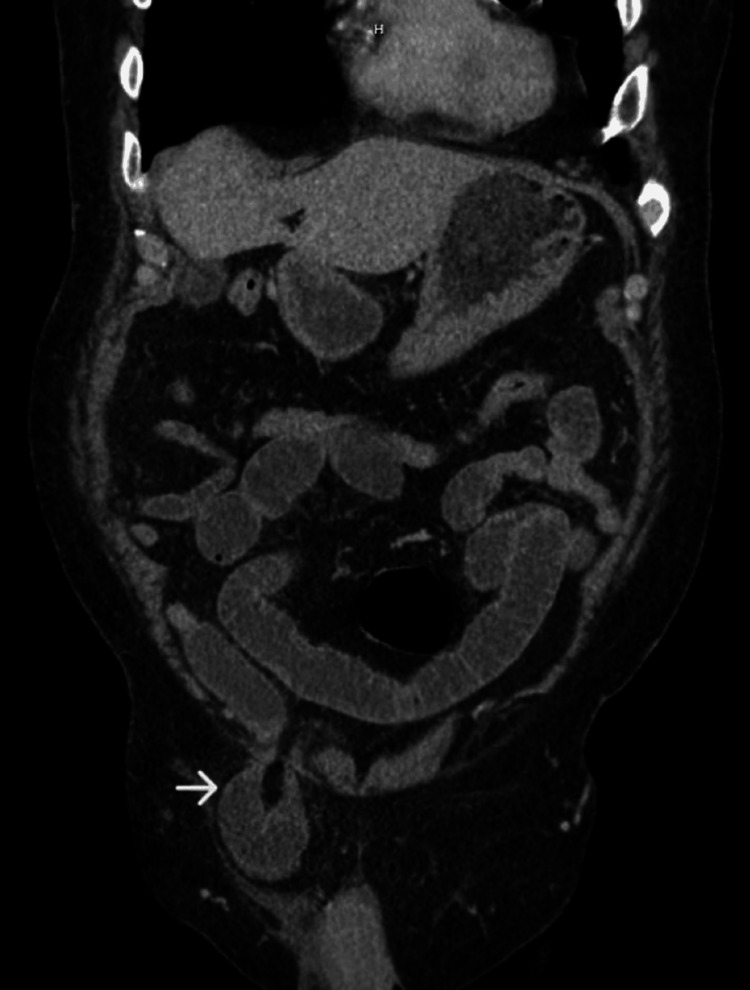
A CT scan image showing a right inguinal hernia containing a bowel loop (→)

**Figure 2:**
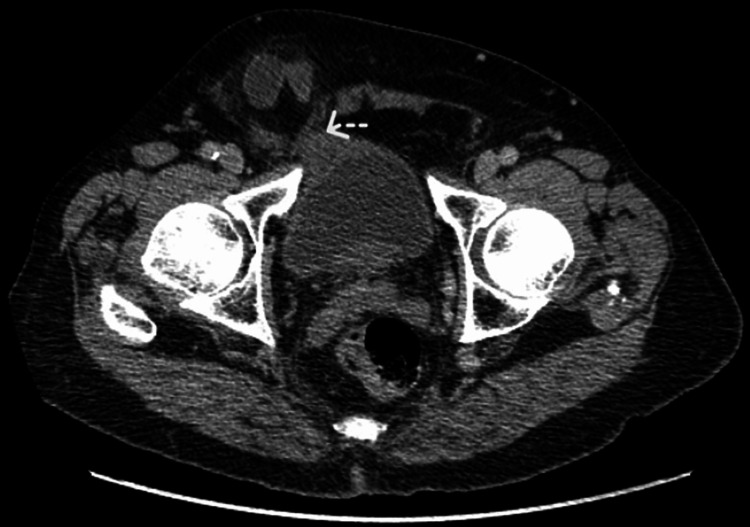
A CT scan image showing a urinary bladder adherent to the hernia sac (→)

An 81-year-old male with a medical history of atrial fibrillation on apixaban and a past surgical history of appendectomy presented to the emergency department with a two-day history of abdominal pain, distension, and swelling in the right groin. He did not report nausea, vomiting, fever, or a change in bowel habits. Physical examination was remarkable for incarcerated right inguinal hernia with tenderness to palpation. A CT scan demonstrated a right inguinal hernia containing a small bowel loop with a diameter measuring 2.5 cm with proximally dilated small bowel loops suggestive of obstruction (Figure 1), in addition to a urinary bladder attached to the medial wall of the sac (Figure 2). Anticoagulation was reversed. Nasogastric tube decompression was initiated. The patient received IV fluid resuscitation, and IV antibiotics, had a Foley catheter placed, and was then urgently taken to the operating room. Under general anesthesia, a right inguinal incision was made. A large direct inguinal hernia containing a small bowel was identified. The herniated bowel was inspected and noted to be healthy without ischemic changes. The bowel was reduced into the abdominal cavity. The hernia sac was identified and found to adhere to the urinary bladder medially. The bladder was dissected off the sac without any injury. The hernia was repaired using a nonabsorbable mesh using the Lichtenstein technique. The postoperative course was uneventful. No hematuria was noted. The Foley catheter was removed on postoperative day one, and he voided spontaneously and without difficulty. He tolerated the diet and had normal bowel movements. The patient was sent home in stable condition postoperatively on day two and was doing well when seen during his postoperative clinic visit approximately two weeks later.

## Discussion

Inguinal hernia is one of the common presentations observed in general surgery practice. The most common contents of a hernia in the inguinal region are the omentum and small bowel. Rarely can it contain unique contents like the appendix, sigmoid colon, cecum, ovary with fallopian tubes, and/or urinary bladder. Inguinal hernias involving the bladder account for less than 4% of all cases.

**Figure 3:**
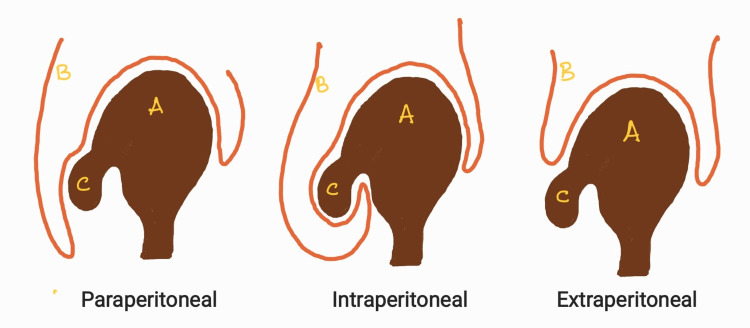
Types of inguinal bladder hernias A: Urinary bladder, B: Peritoneum, C: Hernia

Bladder hernias are classified as paraperitoneal, intraperitoneal, and extraperitoneal based on the relationship of the hernias to the peritoneum (Figure 3) [5]. The paraperitoneal hernia is the most frequent bladder herniation, wherein the bladder remains extraperitoneal and is medial to the hernia sac. In the intraperitoneal hernia, the bladder is covered with the peritoneum in the hernia sac. In an extraperitoneal hernia, only the bladder herniates with no relationship to the peritoneum.

Pathophysiological factors associated with bladder hernias include obesity, bladder outlet obstruction, decreased bladder tone, and weak pelvic muscles [3]. Herniations can range from a small protrusion to an enormous one. Hernias become symptomatic as their size increases. Most patients with inguinoscrotal bladder hernias have two-stage micturition, initiated by spontaneous bladder emptying and carried out by manual compression [3]. There may be nonspecific urinary symptoms, such as frequency, urgency, nocturia, dysuria, or hematuria, associated with bladder outlet obstruction or infection. Various radiologic techniques can be used to establish a diagnosis, including cystography, intravenous pyelography (IVP), CT scans, and ultrasonography. Cystography can reveal a dumbbell-shaped bladder [6]. In their study, Casas et al. described the following diagnostic triad of intravenous urography consisting of lateral displacement of the distal one-third of one or both ureters, a small bladder, and incomplete visualization of the base of the bladder [6]. Additionally, ultrasound can be used to distinguish a bladder from other intrascrotal conditions such as hydrocele, spermatocele, epididymal cysts, and abscesses. In patients with gross hematuria, flexible cystoscopy can be performed to confirm the diagnosis and exclude other pathologies [7]. CT imaging can be used to further demonstrate bladder asymmetry with an extension of a portion of the bladder into the scrotum via the inguinal canal [6].

IBH management includes reducing or resecting the herniated bladder component along with standard repair of the inguinal hernia. The resection of the bladder is indicated for findings of necrosis, a small hernia neck usually less than 0.5 cm, diverticulum of the bladder, or a bladder tumor in the herniated portion [8-10]. Identifying the vesicoureteral junction when resecting the bladder to minimize ureteral injury is important.

## Conclusions

IBHs are uncommon. Unrecognized bladder hernias can cause bladder injury during surgery. High-risk patients including obese, older men, who have urinary symptoms need further preoperative evaluation with a CT scan, ultrasound, or cystography to prevent iatrogenic injury and complications. The herniated bladder should be reduced or resected along with hernia repair. Complications including infarction, necrosis, or obstruction of the bladder outlet can be avoided by not delaying the repair. A general surgeon and urologist must collaborate to manage complicated cases.
